# A Practical Treatise on the Diseases of Children

**Published:** 1846-09

**Authors:** 


*A Practical Treatise on the Diseases of Children.* By JAMES MILMAN COLEY, M. D. etc. etc. ED. BARRINGTON & GEO. D. HASWELL, Philadelphia, 1846; pp. 414, 8vo.

THE above work constitutes the July No. of the Select Medical Library, and is worth a whole year's subscription to that publication. The author, in the introduction, says, "The principal object I have had in view in writing the following pages, has been to present to the medical profession and the public a comprehensive work on the diseases of infants and children, which the physician, the surgeon, and the general practitioner may consult as a work of reference. The practical portions have been the result of an experience of forty years, during which time I have been constantly engaged in recording cases, accumulating facts, and pursuing pathological inquiries. In addition to the various information afforded by my private practice, the numerous and interesting diseases occurring among the poor of an extensive district, to whom I was in the habit of giving gratuitous advise and assistance, either at my own residence, or at an infirmary and dispensary which I established in my native town, supplied me with opportunity rarely enjoyed for prosecuting the useful profession, to which my whole life has been devoted."

We have neither space, nor is it the design of the editors of the American Journal of Dental Science to furnish their readers with an elaborate review or extented notice of the various works on general medicine that are constantly issuing from the press. But impressed with the importance of a more thorough knowledge of the science of disease, than is generally possessed by the practitioners of the dental branch of medicine, they believe it due both to themselves and subscribers, to announce the publication of such works as come under their immediate observation, and to speak in general terms of their respective merits. Hitherto, we have been too remiss in this matter, but for the future we shall endeavor to make amends for our past remissness.

The Select Medical Library is published quarterly; each number contains the republication of an entire work on some branch of medicine. It is edited by John Bell, M. D., of Philadelphia, who is as distinguished a medical writer as he is an eminent practitioner. In connection with the Medical Library, is published monthly the Bulletin of Medical Science, edited by the same gentleman, and both are furnished to subscribers at the very low price of five dollars per annum.—*Balt. Ed.*

